# Contrasting life histories contribute to divergent patterns of genetic diversity and population connectivity in freshwater sculpin fishes

**DOI:** 10.1186/s12862-018-1171-8

**Published:** 2018-04-11

**Authors:** Song Yi Baek, Ji Hyoun Kang, Seo Hee Jo, Ji Eun Jang, Seo Yeon Byeon, Ju-hyoun Wang, Hwang-Goo Lee, Jun-Kil Choi, Hyuk Je Lee

**Affiliations:** 1 Molecular Ecology and Evolution Laboratory, Department of Biological Science, Sangji University, Wonju, South Korea; 2 Korean Entomological Institute, College of Life Sciences and Biotechnology, Korea University, Seoul, South Korea; 3 Molecular Ecology and Evolution Laboratory, Department of Animal Science and Technology, Sangji University, Wonju, South Korea; 4 Animal Ecology Laboratory, Department of Biological Science, Sangji University, Wonju, South Korea

**Keywords:** Cottidae, Dispersal capacity, Freshwater adaptation, Larval stage, Life history, Population genetics, Sculpin, Speciation

## Abstract

**Background:**

Life history characteristics are considered important factors influencing the evolutionary processes of natural populations, including the patterns of population genetic structure of a species. The sister species *Cottus hangiongensis* and *C. koreanus* are small bottom-dwelling freshwater sculpin fishes from South Korea that display marked life history divergence but are morphologically nearly indistinguishable. *Cottus hangiongensis* evolved an ‘amphidromous’ life history with a post-hatching pelagic larval phase. They spawn many small eggs in the low reaches of rivers, and hatched larvae migrate to the sea before returning to grow to maturity in the river mouth. In contrast, *C. koreanus* evolved a ‘fluvial’ landlocked type with benthic larvae. They release a smaller number of larger eggs, and the larvae undergo direct development, remaining benthic in the upstream rivers throughout their entire lives. We tested whether there were differences in patterns and levels of within-population genetic diversities and spatial population structure between the two closely related Korean sculpins using mitochondrial DNA control region sequences and seven nuclear microsatellite loci.

**Results:**

The combined analyses of both marker sets revealed that *C. hangiongensis* harboured considerably higher levels of within-population genetic diversities (e.g. haplotype/allelic richness, heterozygosities) than *C. koreanus*. In contrast, the fluvial sculpin exhibited noticeably more spatial population structure than did the amphidromous sculpin, as suggested by pairwise *F*_ST_ statistics. The finding that *C. hangiongensis* individuals comprised a single random mating population across the east-flowing river basins in the Korean Peninsula, whereas *C. koreanus* individuals comprised genetically discrete individual populations, was further supported by an individual-based Bayesian population assignment and also factorial correspondence analyses.

**Conclusions:**

The higher genetic diversity, but lower population structure, of the amphidromous sculpin relative to the fluvial sculpin may have resulted from its greater larval dispersal and also possibly, higher fecundity accompanied by an amphidromous life history. Hence, we conclude that contrasting early life histories – including the presence or absence of the pelagic larval phase – may have led to divergent patterns of within-population genetic diversities and spatial population structure between the sister *Cottus* species following speciation from a common ancestor of marine sculpin.

**Electronic supplementary material:**

The online version of this article (10.1186/s12862-018-1171-8) contains supplementary material, which is available to authorized users.

## Background

Life history traits are considered important biological factors affecting the evolutionary processes, as they shape the patterns of population genetic structure of a species [1–7]. An organism’s life history can be defined as a set of age- or stage-specific traits that contribute to survival and/or reproduction (i.e. fitness), upon which selection acts [8]. Life history attributes can influence population connectivity as they constrain the movement of individuals (i.e. dispersal), which affects the distribution of within- and among-population genetic variation and contributes to the present-day population genetic structure of a species [3–5]. Dispersal is, therefore, thought to play a pivotal role in population dynamics, colonization of novel habitats and geographic distribution of freshwater and marine fishes [9]. In this regard, the presence or absence of a post-hatching planktonic larval period at an early life history stage has been suggested to be a key component in characterizing the degree and pattern of population connectivity or geographic population structure in both marine [10, 11] and freshwater species [3, 5, 6, 12].

A number of previous studies suggest that life history characteristics are significantly associated with the degree of population connectivity of freshwater fishes [6, 13]. In salmonid fishes that have evolved and diverged into migratory and resident populations within species, the greater dispersal capacity of migratory populations has generally resulted in lower degrees of spatial population structure relative to resident populations [14]. Although several studies have been undertaken to understand the impacts of adult movement (i.e. migratory behaviour) on levels of genetic divergence among freshwater fish populations [4, 7], the effects of life history divergence with a focus on the presence or absence of the post-hatching larval phase on the genetic structure of freshwater fishes have not been widely tested (but see [3, 5]). Therefore, studying evolutionarily closely related species with contrasting life history types over small geographic scales will provide a good opportunity for directly testing the influences of the larval phase on population connectivity. Moreover, it will allow us to determine the role of life history in shaping the patterns of intraspecific genetic variation in light of the evolution of geographic population structure [6].

Freshwater fishes have evolved an extraordinarily diverse array of life history characteristics. The best reported example of the divergent life histories between populations or closely related species of freshwater fishes comes from the salmonid fishes [13]. For instance, Chinook salmon (*Oncorhynchus tshawytscha*) is characterized by two distinct life history types with different patterns of geographic distribution as ‘ocean-type’ and ‘stream-type’. These life history types are distinguished based on the duration that juvenile salmon remain in freshwater habitats before migrating to the sea [15, 16]. Another case of the diverse life history types among closely related freshwater fish species includes the genus *Rhinogobius*, in which species with or without a pelagic larval phase exist [17, 18].

The freshwater sculpins of the genus *Cottus* are bottom-dwelling fishes that comprise approximately 68 species from the subarctic areas to the temperate northern hemisphere, including Europe, Siberia, Central and East Asia and North America [19–22]. These typically cold-water fishes are believed to have originated several times in parallel from different ancestral species of marine sculpins [20]. Interestingly, within the genus *Cottus*, four distinct life history styles have evolved, including marine, catadromous, amphidromous, and fluvial (landlocked/lacustrine) types [20, 23].

**Fig. 1:**
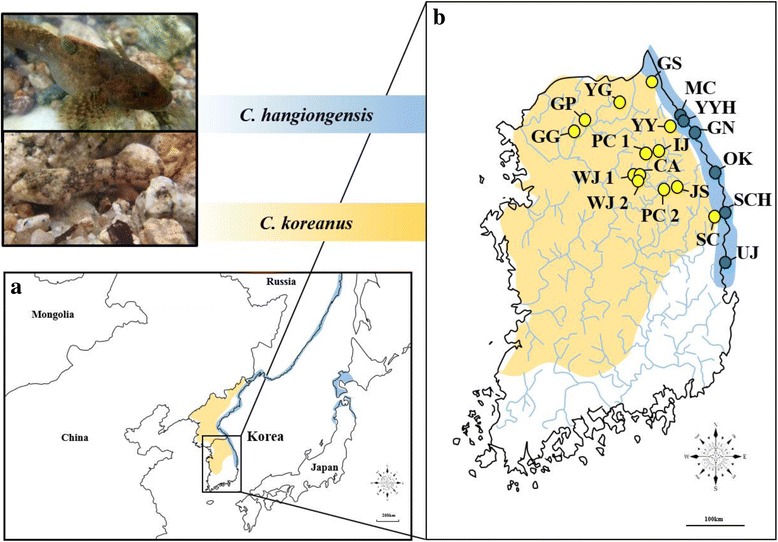
The geographic distribution and sampling localities of *C. hangiongensis* and *C. koreanus*. **a** The entire geographical ranges of both species (*C. hangiongensis* in blue and *C. koreanus* in yellow). **b** Within South Korea, the geographic distribution of *C. hangiongensis* is limited to the east-flowing rivers (shaded areas in blue), but that of *C. koreanus* ranges from the Han River further south to the Nakdong River in inland areas (in yellow). Circles in blue denote sampling sites for *C. hangiongensis* (*N* = 6) and those in yellow indicate sampling localities for *C. koreanus* (*N* = 13). The latitude and longitude of each sampling location is shown in Additional file 1: Table S1. Population abbreviations as in text and Additional file 1: Table S1

The sister species *C. hangiongensis* and *C. koreanus* [23] have contrasting life histories and differ in reproductive behaviour and geographic distribution, but are morphologically virtually indistinguishable as adults [24]. *Cottus hangiongensis* shows an amphidromous life history, where they spawn large numbers of small eggs (752–1376 per clutch) in the lower courses of rivers during early spring [24]. The hatched pelagic larvae then migrate to the sea by river flows. After the pelagic larvae spend approximately 4–6 weeks in marine environments, the juveniles return to rivers to grow and then maintain a benthic life in downstream rivers [25, 26]. Whether the juveniles of *C. hangiongensis* return to the same rivers where they hatched, however, remains unknown. On the other hand, *C. koreanus* shows a fluvial life history, where they produce relatively fewer, but larger eggs (538–880 per clutch), and the larvae undergo direct development, remaining benthic in the upstream rivers immediately after hatching [24, 27]. For spawning, although mature individuals of *C. hangiongensis* migrate downstream to the spawning ground [27], *C. koreanus* individuals do not move and stay at their natal habitats [28]. These reproductive behaviours, however, imply that both species remain in the same rivers throughout their entire lives as adults. Males of these species usually mate continuously with several females and remain in the nest after spawning (i.e. polygyny). The migration ranges of settled adult individuals are estimated to be around 10 m for *C. koreanus* [28] and about 100 m for *C. hangiongensis* [29], which suggests restricted dispersal capacities for both species at an adult life history phase. *Cottus hangiongensis* is distributed across the eastern Korean Peninsula, northern Japan and Russia, whereas *C. koreanus* is endemic to the Korean Peninsula [30] (Fig. 1a). Within South Korea, *C. hangiongensis* occurs in rivers that flow into the East Sea from central to northern parts of eastern Korea, and *C. koreanus* is distributed in the Han River, Imjin River, and Nakdong River in inland regions [31] (Fig. 1b). In these Korean sculpins, speciation from a common ancestor of marine sculpin was accompanied by ecological divergence into amphidromous and fluvial life histories, as seen for another group of Japanese sculpins, *C. amblystomopsis* and *C. nozawae* [32, 33]. The divergence time between *C. hangiongensis* and *C. koreanus* has been inferred from a mitochondrial DNA (mtDNA) phylogeny to be approximately 1.7–4.0 Mya [23]. *Cottus hangiongensis* populations in South Korea have experienced recent sharp declines because they are susceptible to changes in water environments caused by anthropogenic pressure, such as agricultural run-off, water pollution by sewage disposal, and impoundments for water retention [34, 35]. Therefore, this species has been protected by the Korean government as a ‘legally protected species II’ since 2012 (endangered wild species class II), but *C. koreanus* has recently been removed from the Red List of Korean endangered species.

Several studies on Korean populations of *C. hangiongensis* and/or *C. koreanus* have been performed to understand their morphology [24], reproductive and spawning behaviours [27, 36], feeding ecology [37, 38], and capacity for natural hybridization [31, 39]. Previous morphological surveys suggest natural hybridization is possible between these two species [31, 39]. However, little attention has been paid to the population genetic structure of Korean populations of both species (but see [39]). In particular, the potential role of life history in shaping the population genetic structure of this species pair remains largely unexplored.

In this study, we examined whether there were differences in patterns and levels of genetic diversity and population connectivity, as well as geographic population structure between the amphidromous species *C. hangiongensis*, which has a pelagic larval stage, and the fluvial species *C. koreanus*, which has direct development. To this end, we used mtDNA control region sequences and seven nuclear microsatellite loci to analyse and compare the levels of within-population genetic diversity among five populations of *C. hangiongensis* and 10 populations of *C. koreanus* in South Korea. Due to its greater capability to disperse, *C. hangiongensis* is expected to show much lower levels of geographic population structure, but a higher extent of within-population genetic diversity than *C. koreanus*. The results of this study will advance our understanding of how life history features influence the population structure and also provide conservation implications for these endangered species.

## Methods

### Study sites and sample collection

Amphidromous *C. hangiongensis* (*N* = 225) and fluvial *C. koreanus* (*N* = 273) were sampled from six and 13 different river drainages (localities), respectively, using skimming nets in South Korea between 2014 and 2016 (Fig. 1b; Table 1; Additional file 1: Table S1). The sampling sites for *C. hangiongensis* (Fig. 1b) included the downstream regions of the east-flowing rivers in the Korean Peninsula, such as Uljin from the Wangpi Stream (UJ), Samcheok from the Osip Stream (SCH), Yangyang from the Namdae Stream (YYH), Yangyang from the Mulchi Stream (MC), Gangneung from the Yeongok Stream (GN) and Okgye from the Nakpung Stream (OK). Those for *C. koreanus* (Fig. 1b) included Pyeongchang from the Heungjeong Valley (PC 1), Pyeongchang from the Gihwa Stream (PC 2), Jeongseon (JS), Wonju Oakvalley (WJ 1), Wonju from the Gangrim Stream (WJ 2) and Wonju from the Bugok Valley at Chiaksan National Park (CA) from the South Han River; Goseong from the Baebong Stream (GS), Yanggu (YG), Inje from the Bangtae Stream (IJ), Gapyeong from the Jojong Stream (GP) and Pocheon (GG) from the North Han River; Samcheok from the Osip Stream (SC) and Yangyang from the Namdae Stream (YY), which are the east-flowing rivers where *C. hangiongensis* occurs downstream rivers. Some of our sample sizes (*C. hangiongensis*: OK; *C. koreanus*: WJ 1, GS, YG) were small (*N* < 10) because of the recent sharp declines of these particular populations (S. Y. Baek, personal observation). A small piece (approximately 3 mm) of caudal fin tissue was collected from each individual (all individuals of *C. hangiongensis* collected were then released back to the original sites), and preserved immediately in 99% ethanol and stored at 4 °C until genetic analysis. Field collection of *C. hangiongensis* was conducted under a study permit (No: 2014–7, 2014–08, 2015–17, 2014’ Park Conservation Department-1054, 2016’ Park Conservation Department-893) granted by the Regional Environmental Offices of the South Korean government and the Korea National Park Service.

**Table 1:** Summary of the level of genetic diversity in six and 13 geographic populations of *C. hangiongensis* and *C. koreanus* in South Korea at both mtDNA control region and seven microsatellite loci

	Population	MtDNA						Microsatellites							
		*N*	*N*_H_	HR	PH	*h*	*π*	*N*	*N*_A_	AR	PA	*H*_E_	*H*_O_	*F*_IS_	H-W tests (*P*)
*C. hangiongensis*	SCH	82	7	3.434	4	0.417	0.001	51	16.57	13.41	7	0.800	0.769	0.040	**
	YYH	42	3	1.588	0	0.257	0.001	31	15.29	13.80	3	0.796	0.751	0.058	NS
	UJ	41	4	1.935	0	0.474	0.001	31	13.29	11.83	2	0.690	0.687	0.004	NS
	GN	34	3	2.602	0	0.355	0.001	35	14.43	12.62	6	0.772	0.763	0.011	NS
	MC	25	3	2.000	0	0.353	0.001	26	15.00	14.49	5	0.799	0.802	−0.004	NS
	OK	1	1	–	0	–	–	–	–	–	–	–	–	–	–
	Total	225	8	–	4	0.379	0.001	174	22.43	14.31	23	0.788	0.755	–	**
*C. koreanus*	PC 1	25	6	4.881	4	0.770	0.015	26	8.29	7.69	9	0.726	0.696	0.042	**
	PC 2	25	5	3.710	4	0.710	0.003	26	11.14	10.42	10	0.811	0.835	−0.033	NS
	WJ 1	9	1	–	0	–	–	9	2.86	–	1	–	–	–	–
	WJ 2	25	1	0.000	0	0.000	0.000	25	5.14	4.89	0	0.523	0.543	−0.039	NS
	CA	23	1	0.000	1	0.000	0.000	24	3.29	3.25	0	0.492	0.511	−0.039	NS
	JS	24	4	2.787	4	0.605	0.003	25	9.57	9.13	9	0.766	0.717	0.060	**
	SC	34	3	1.941	3	0.399	0.002	20	2.71	2.71	4	0.289	0.307	−0.065	NS
	YY	19	1	0.000	1	0.000	0.000	20	2.14	2.14	3	0.223	0.286	−0.291	NS
	GS	7	1	–	1	–	–	8	5.57	–	22	–	–	–	–
	YG	2	1	–	1	–	–	2	1.71	–	2	–	–	–	–
	IJ	23	1	0.000	1	0.000	0.000	23	2.00	1.95	2	0.197	0.250	−0.280	**
	GP	27	1	0.000	0	0.000	0.000	27	3.86	3.77	3	0.575	0.683	−0.192	**
	GG	30	1	0.000	0	0.000	0.000	25	4.57	4.27	9	0.514	0.486	0.056	**
	Total	273	24	–	20	0.905	0.019	260	25.86	13.02	74	0.896	0.546	–	**

*N* sample sizes, *N*_H_ number of haplotypes, *HR* haplotype richness, *PH* number of private haplotypes, *h* haplotype diversity, *π* nucleotide diversity, *N*_A_: observed mean number of alleles across seven loci, *AR* allelic richness, *PA* number of private alleles, *H*_E_ expected heterozygosity, *H*_O_ observed heterozygosity, *F*_IS_ inbreeding coefficient, and H-W tests (*P*): *P* values for multi-locus tests for Hardy Weinberg equilibrium (HWE). **: *P* < 0.01 after a Bonferroni correction applied, NS: not significant

Four populations (*C. hangiongensis*: OK, *C. koreanus*: WJ 1, GS and YG) with insufficient sample sizes (*N* < 10) were excluded from some of the genetic diversity statistics. Population abbreviations as in text and Additional file 1: Table S1

### MtDNA control region sequencing

Genomic DNA was isolated using a DNeasy Blood and Tissue Kit (Qiagen, Hilden, Germany). A 465- and a 467-bp sequence of the mtDNA control regions (CR) of *C. hangiongensis* and *C. koreanus*, respectively, were amplified using the published forward and reverse primers L-Thr, H12Sr5 and internal primer LCCR [40]. Polymerase chain reaction (PCR) amplification was performed in a reaction volume of 15 μl comprising 1× PCR buffer, 25 μM of each dNTP (Bio Basic Inc., Markham, ON, Canada), 0.6 μM of each of the forward and reverse primers and 0.2 U of *Taq* polymerase (Thermo Fisher Scientific, Waltham, MA, USA). The following thermal cycling conditions were used: initial denaturation at 94 °C for 1 min followed by 35 cycles of denaturation at 94 °C for 1 min, annealing at 58 °C for 1 min and extension at 72 °C for 1 min, followed by a final extension at 72 °C for 20 min in a 2720 thermal cycler (Applied Biosystems, Foster City, CA, USA). PCR products were checked on 2% agarose gels stained with RedSafe (iNtRon Biotechnology, Daejeon, Korea). The amplified PCR products were purified enzymatically with Exonuclease I (New England BioLabs, Ipswich, MA, USA) and Shrimp Alkaline Phosphatase (New England BioLabs). The purified mtDNA fragments were subjected to direct sequencing using the internal forward LCCR and the same reverse H12Sr5 primers as in the PCR, and the BigDye Terminator 3.1 Cycle Sequencing Ready Reaction Kit in an ABI 3730xl automated DNA sequencer (Applied Biosystems). The DNA sequences were edited using CHROMAS v2.01 computer software and aligned with Clustal Omega [41], implemented in BioEdit v7.2.5 [42], and finally verified by visual inspection.

### Microsatellite genotyping

The seven published primer pairs, which were originally developed for the European bullhead species *Cottus gobio* [43, 44], were used for microsatellite genotyping of our study species. Seven polymorphic nuclear microsatellite loci were chosen, including Cgo56, Cgo05, Cgo18, Cgo22 [43], Cott138, Cott207 and Cott112 [44]. The forward primers were labelled with fluorescent dye (FAM, NED, VIC and PET). PCR reactions were carried out as described for amplifying the mtDNA control region. PCR cycling conditions comprised an initial denaturation phase at 94 °C for 1 min, 35 cycles of 94 °C for 1 min, 57 °C–64 °C for 1 min, 72 °C for 1 min and a final extension step at 72 °C for 20 min. The PCR products were electrophoresed on an ABI 3730xl automated DNA sequencer (Applied Biosystems). Fragment sizes were determined to a ROX 500-bp size standard (ABI), as detected using GENEMAPPER software v5.0 (Applied Biosystems).

### Statistical analyses

#### Genetic diversity

To determine levels of mitochondrial diversity in both *C. hangiongensis* and *C. koreanus*, the numbers of polymorphic sites and haplotypes, haplotype diversity (*h*) and nucleotide diversity (*π*) were estimated for each population, as well as for the entire species using ARLEQUIN v3.5 [45]. A rarefaction method was applied using CONTRIB v1.02 [46] to estimate haplotype richness (HR), corrected for unequal sample sizes among the samples. The HR estimates were calculated after excluding the samples of OK (*N* = 1) for *C. hangiongensis*, and WJ 1 (*N* = 9), GS (*N* = 7) and YG (*N* = 2) for *C. koreanus*, because of insufficient sample sizes (Table 1). These four samples were omitted from downstream population genetic analyses, with the exception of the mtDNA haplotype network and microsatellite-based STRUCTURE and factorial correspondence analyses (FCA). The OK sample was omitted from STRUCTURE and FCA analyses because it was comprised of only a single individual. To investigate relationships among the mtDNA haplotypes of each species, a haplotype network was constructed with HAPSTAR v0.7 [47], based on the minimum spanning tree generated from ARLEQUIN.

To assess microsatellite diversity in each species, mean number of alleles per locus (*N*_A_), expected (*H*_E_) and observed (*H*_O_) heterozygosity, inbreeding coefficient (*F*_IS_), and allelic richness (AR) corrected for unequal sample sizes were estimated using GENEPOP v4.0 [48] and FSTAT v2.9.3.2 [49]. The presence of null alleles was assessed using MICROCHECKER v2.2.3 [50] with 1000 randomizations at the 95% confidence level. Genotypes at the seven microsatellite loci were tested for linkage disequilibrium (LD) separately for each species, and multi-locus tests for Hardy-Weinberg equilibrium (HWE) were undertaken for every sample of each species using GENEPOP. The 95% significance levels for every exact test for both LD and HWE were adjusted using a Bonferroni correction.

A recent reduction in effective population sizes (*N*_e_) was tested using BOTTLENECK v1.2.02 [51], by applying 10,000 permutations for the two-phase mutation (TPM) model of microsatellite evolution. The TPM model was used because it has been shown to be the best fitting for a microsatellite dataset [51] and allows for multi-step mutations. A Wilcoxon sign-rank test was used to determine whether the populations of both species showed a significant heterozygosity excess, which would be expected with founder effects or recent population bottlenecks [52]. Contemporary *N*_e_ was also calculated for each of the samples in both species according to the LD method implemented in NEESTIMATOR v2.01 [53].

Two independent Mann-Whitney *U* tests were conducted to test whether there were significant differences in the levels of mitochondrial (HR) and microsatellite (AR) diversities between populations of *C. hangiongensis* (*N* = 5) and *C. koreanus* (*N* = 10). The significant differences in levels of *H*_E_ and *H*_O_ between the two species were also statistically analysed, as done for HR and AR.

#### Spatial population genetic structure

To examine the spatial genetic differentiation between populations within each species, exact tests for population differentiation [54], as well as calculation of pairwise estimates of *F*_ST_ [55] at both markers, were carried out using ARLEQUIN and GENEPOP, respectively. The 95% significance levels for the pairwise population comparisons were adjusted using a Bonferroni correction. In addition, we tested for isolation by distance (IBD) among samples separately for each species at both markers using the Mantel test in GENALEX v6.502 [56]. The geographic surface distance in kilometres between two sampling sites was obtained from the website (http://www.movable-type.co.uk/scripts/latlong.html), based on the coordinate information (latitude/longitude) for each location.

We further analysed the spatial population genetic structure of each species using an individual-based Bayesian population assignment test in STRUCTURE v2.3.1 [57], under an admixture model, and correlated allele frequencies with no a priori information on the geographic origins of the samples. We tested 10 iterations at each *K* = 1–18 (*C. hangiongensis*: number of samples [*N*] = 5; *C. koreanus*: *N* = 13), with 50,000 burn-in steps followed by 500,000 Markov chain Monte Carlo generations. The most probable number of clusters (*K* value) was estimated using the ΔK method implemented in the web-based tool Structure Harvester (http://taylor0.biology.ucla.edu/structureHarvester/) [58], based on the rate of change in the log probability of data between successive *K* values. STRUCTURE analyses were also performed separately for populations of *C. hangiongensis* and *C. koreanus*. Finally, FCA, based on genetic relationships among individuals with multi-locus genotypes, was performed for both species using GENETIX v4.05.2 [59].

## Results

### Levels of genetic diversity between *C. hangiongensis* and *C. koreanus*

**Fig. 2:**
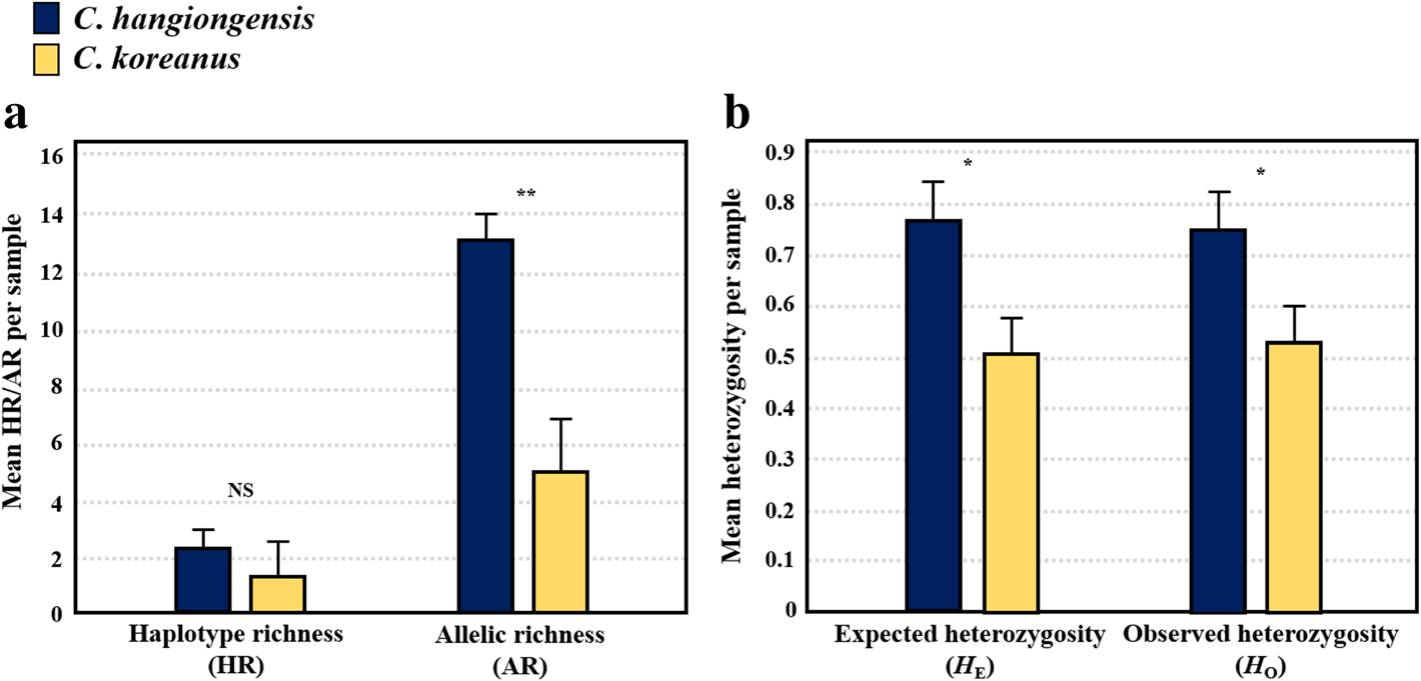
Comparisons of the level of genetic diversity between *C. hangiongensi*s and *C. koreanus*. **a** Average mtDNA haplotype richness (HR) and microsatellite allelic richness (AR) per sample; **b** Average observed (*H*_O_) and expected (*H*_E_) heterozygosity per sample. Levels of AR, *H*_O_ and *H*_E_ (except HR) were significantly higher (*P* < 0.05) for *C. hangiongensis* than for *C. koreanus*. *: *P* < 0.05; **: *P* < 0.01; NS: not significant

**Fig. 3:**
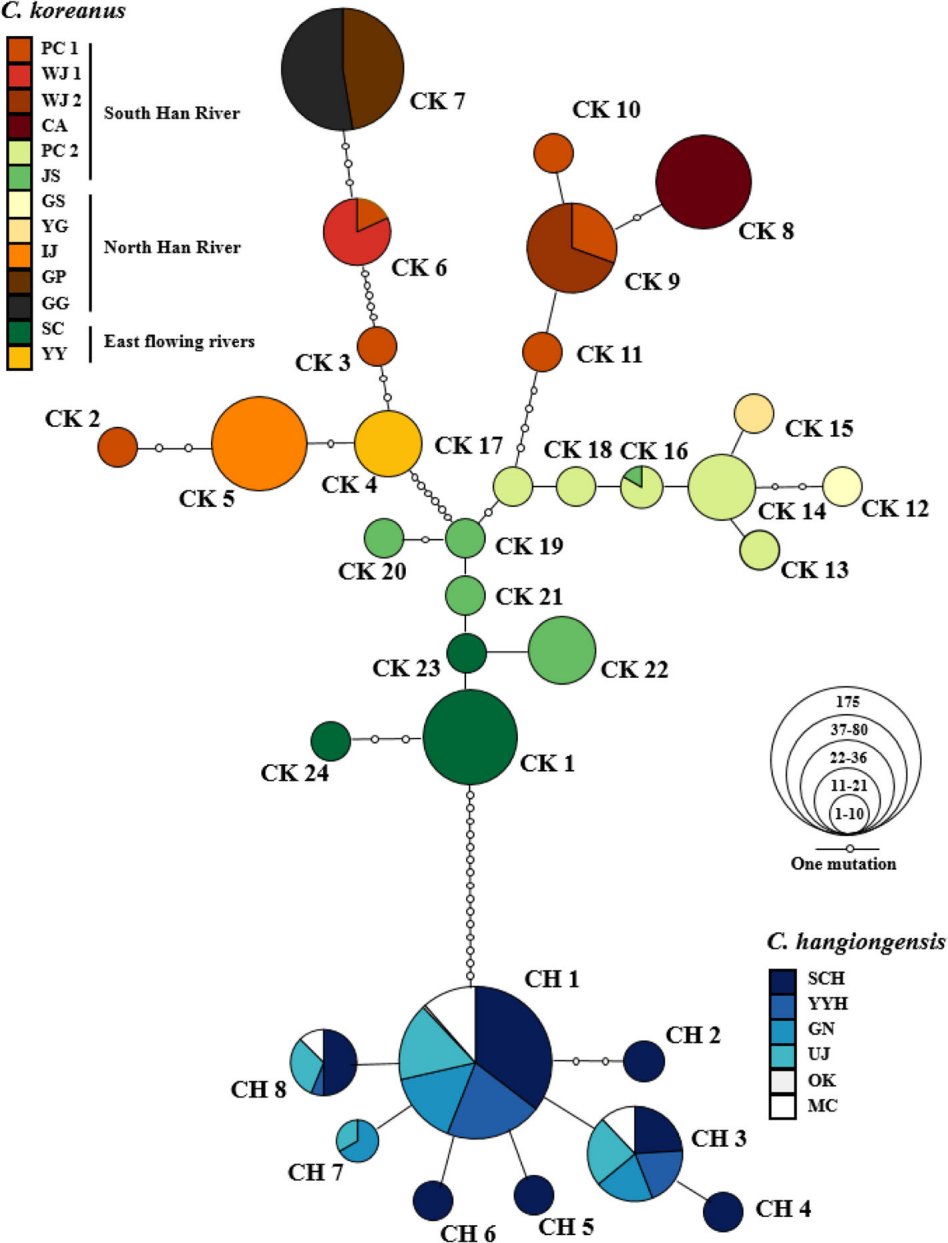
Haplotype networks of *C. hangiongensis* and *C. koreanus* based on mtDNA control region (465, 467 bp each). A total of eight and 24 haplotypes were detected in *C. hangiongensis* and *C. koreanus*, respectively. The size of the circle is proportional to number of individuals that belong to the respective haplotypes and small white circles denote intermediate haplotypes that are not present in our samples, but are necessary to connect all of the observed haplotypes to the network. Each node in the network represents a single mutational step between haplotypes irrespective of its length. Population abbreviations as in text and Additional file 1: Table S1

Levels of within-population genetic diversity were higher for the amphidromous species *C. hangiongensis*, than for the fluvial species *C. koreanus* (Fig. 2; Table 1). A total of eight mtDNA haplotypes (CH 1–8: GenBank Accession Nos. MF405328–MF405335) were identified in 225 individuals from six populations of *C. hangiongensis*, whereas 24 haplotypes (CK 1–24: GenBank Accession Nos. MF405304–MF405327) were found in 273 individuals from 13 populations of *C. koreanus* (Fig. 3; Table 1). While four out of eight mtDNA haplotypes (CH 1, 3, 7 and 8) in *C. hangiongensis* were shared by 2–6 populations, only four of 24 haplotypes (CK 6, 7, 9 and 16) in *C. koreanus* were shared between two populations, and the remaining 20 haplotypes were private haplotypes found in a single population (Fig. 3). The overall values of *h* and *π* for *C. hangiongensis* and *C. koreanus* were 0.379 ± 0.039 and 0.001 ± 0.001, and 0.905 ± 0.008 and 0.019 ± 0.011, respectively (Table 1). The HR values ranged from 1.588 (YYH) to 3.434 (SCH) (mean = 2.312) for *C. hangiongensis* and from 0.000 (WJ 2, CA, YY, IJ, GP, GG) to 4.881 (PC 1) (mean = 1.332) for *C. koreanus* (Mann-Whitney *U*, *P* = 0.31; Fig. 2a). The haplotype networks of *C. hangiongensis* and *C. koreanus* could be connected by sixteen mutational steps (*C. hangiongensis*–CH 1 to *C. koreanus*–CK 1), and no haplotypes were shared between the two species (Fig. 3). For *C. hangiongensis*, the most common haplotype, CH 1, was predominant across all populations, with a frequency of 78% (175 out of 225 individuals).

The levels of microsatellite diversity (AR) were significantly higher in amphidromous *C. hangiongensis* than in fluvial *C. koreanus* (*C. hangiongensis*: mean AR = 13.23; *C. koreanus*: mean = 5.02; Mann-Whitney *U*, *P* < 0.001; Fig. 2a; Table 1). The mean number of alleles across the seven loci (*N*_A_) per population for *C. hangiongensis* was 14.92, ranging from 13.29 (UJ) to 16.57 (SCH), whereas the mean in *C. koreanus* was 4.83, ranging from 1.71 (YG) to 11.14 (PC 2) (Table 1). Similarly, levels of *H*_E_ and *H*_O_ were significantly higher in *C. hangiongensis* (*H*_E_: mean = 0.771; *H*_O_: mean = 0.754) than in *C. koreanus* (*H*_E_: mean = 0.512; *H*_O_: mean = 0.531) (*H*_E_: Mann-Whitney *U*, *P* = 0.03; *H*_O_: *P* = 0.03) (Fig. 2b; Table 1). However, the number of private alleles (PA) detected was approximately 3.2 times higher for *C. koreanus* (*N* = 74) than for *C. hangiongensis* (*N* = 23), despite more populations having been analysed for *C. koreanus*. The highest number of PA was identified in SCH (*N* = 7) for *C. hangiongensis* and in GS (*N* = 22) for *C. koreanus*. The *F*_IS_ values ranged from − 0.004 (MC) to 0.058 (YYH) for *C. hangiongensis* and from − 0.291 (YY) to 0.060 (JS) for *C. koreanus*. Based on our multi-locus tests for HWE expectations, only one population in *C. hangiongensis* (SCH) and five populations in *C. koreanus* (PC 1, JS, IJ, GP and GG) may be experiencing non-random mating (inbreeding or outbreeding) at the seven loci analysed (Table 1). The estimated frequencies of null alleles at the seven loci were close to zero, ranging from 0.004 (Cgo18) to 0.094 (Cott112) for *C. hangiongensis* and from 0.048 (Cgo22) to 0.314 (Cott138) for *C. koreanus*, indicating a low probability of null alleles. Tests of LD across the seven loci were not statistically significant (*P* > 0.05) for either species after a Bonferroni correction, suggesting that all loci were independent markers.

BOTTELNECK analysis revealed that *C. hangiongensis* had no sign of population bottlenecks in the five populations analysed (Additional file 1: Table S2). Similarly, only one population (CA) in *C. koreanus* had allelic distribution shifts (mode-shift), which are typically considered to be evidence for population bottlenecks or founder effects (Table S2). However, CA showed no significant heterozygosity excess using a Wilcoxon sign-rank test (*P* = 0.469). The LD method gave median estimates of effective population size (*N*_e_) of 161.2 (95% confidence interval [CI]: 74.3–∞) and an infinite *N*_e_ (95% CL: 686.2–∞) for the GN and SCH populations of *C. hangiongensis*, respectively (Table 2). However, the estimates of *N*_e_ for the populations in *C. koreanus* were generally much lower, ranging from 3.8 (95% CI: 1.3–23.7) for GG to an infinite *N*_e_ (95% CI: 46.6–∞) for GP (Table 2).

**Table 2:** Estimates of contemporary effective population sizes (*N*_e_) for five and 10 populations of *C. hangiongensis* and *C. koreanus*, respectively in South Korea based on linkage disequilibrium (LD) method using NEESTIMATOR v2.01 [53]

Species	Population	Median of *N*_e_	95% confidence interval (CI)
*C*. *hangiongensis*	SCH	infinity	686.2 – infinity
	YYH	717.9	123.2 – infinity
	UJ	576.6	82.1 – infinity
	GN	161.2	74.3 – infinity
	MC	infinity	241.8 – infinity
*C*. *koreanus*	PC 1	11.0	6.3–20.0
	PC 2	64.6	30.3–985.3
	WJ 2	151.9	34.5 – infinity
	CA	infinity	19.5 – infinity
	JS	61.4	28.8–837.5
	SC	6.0	2.3–17.2
	YY	105.9	3.2 – infinity
	IJ	infinity	15.4 – infinity
	GP	infinity	46.6 – infinity
	GG	3.8	1.3–23.7

Population abbreviations as in text and Additional file 1: Table S1

### Degree of spatial genetic structure between *C. hangiongensis* and *C. koreanus*

The degree of spatial genetic differentiation (*F*_ST_) between populations at both mtDNA and microsatellite markers was much lower for the amphidromous species *C. hangiongensis* than for the fluvial species *C. koreanus* (Table 3). The pairwise estimates of *F*_ST_ between five samples of *C. hangiongensis* at mtDNA and microsatellites ranged from − 0.026 to 0.019 and from − 0.002 to 0.050, respectively. Unexpectedly, we observed weak but statistically significant genetic differentiation between samples of *C. hangiongensis* at only microsatellites (except YYH vs MC) (Table 3). The *F*_ST_ values estimated between 10 samples of *C. koreanus* were all highly significant (*P* < 0.01) and considerably higher than those of *C. hangiongensis*, ranging from 0.285 to 1.000 at mtDNA and from 0.065 to 0.761 at microsatellites. The Mantel tests of both species showed a lack of a significant correlation between geographic (km) and genetic (*F*_ST_ values) distances among the populations (mtDNA: *C. hangiongensis*, *r* = 0.01, *P* = 0.50; *C. koreanus*, *r* = 0.31, *P* = 0.45; microsatellites: *C. hangiongensis*, *r* = 0.50, *P* = 0.07; *C. koreanus*, *r* = 0.15, *P* = 0.20).

**Table 3:** Spatial genetic differentiation (as indicated by pairwise estimates of *F*_ST_) between five and 10 populations of *C. hangiongensis* and *C. koreanus*, respectively based on mtDNA control region sequences (below diagonal) and seven microsatellite loci genotypes (above diagonal)

(a)	SCH		YYH		UJ		GN		MC	
SCH			**0.017**		**0.050**		**0.012**		**0.024**	
YYH	0.003				**0.028**		**0.015**		−0.002	
UJ	−0.003		0.003				**0.032**		**0.027**	
GN	0.019		−0.011		0.008				**0.011**	
MC	−0.017		−0.021		−0.026		−0.006			
(b)	PC 1	PC 2	WJ 2	CA	JS	SC	YY	IJ	GP	GG
PC 1		**0.186**	**0.362**	**0.375**	**0.206**	**0.467**	**0.508**	**0.491**	**0.296**	**0.304**
PC 2	**0.537**		**0.262**	**0.287**	**0.069**	**0.416**	**0.424**	**0.450**	**0.294**	**0.325**
WJ 2	**0.285**	**0.928**		**0.065**	**0.279**	**0.542**	**0.615**	**0.632**	**0.450**	**0.464**
CA	**0.347**	**0.940**	**1.000**		**0.300**	**0.530**	**0.629**	**0.643**	**0.464**	**0.491**
JS	**0.537**	**0.741**	**0.919**	**0.893**		**0.433**	**0.469**	**0.492**	**0.312**	**0.352**
SC	**0.635**	**0.735**	**0.948**	**0.934**	**0.557**		**0.745**	**0.761**	**0.543**	**0.590**
YY	**0.577**	**0.941**	**1.000**	**1.000**	**0.897**	**0.936**		**0.555**	**0.540**	**0.620**
IJ	**0.586**	**0.945**	**1.000**	**1.000**	**0.906**	**0.941**	**1.000**		**0.539**	**0.579**
GP	**0.746**	**0.953**	**1.000**	**1.000**	**0.940**	**0.946**	**1.000**	**1.000**		**0.389**
GG	**0.757**	**0.955**	**1.000**	**1.000**	**0.943**	**0.948**	**1.000**	**1.000**	**0.000**	

Significant pairwise *F*_ST_ and *P* values are shown in bold (*P* < 0.001) after the Bonferroni correction. (a) *C*. *hangiongensis*, (b) *C*. *koreanus.* Population abbreviations as in text and Additional file 1: Table S1

**Fig. 4:**
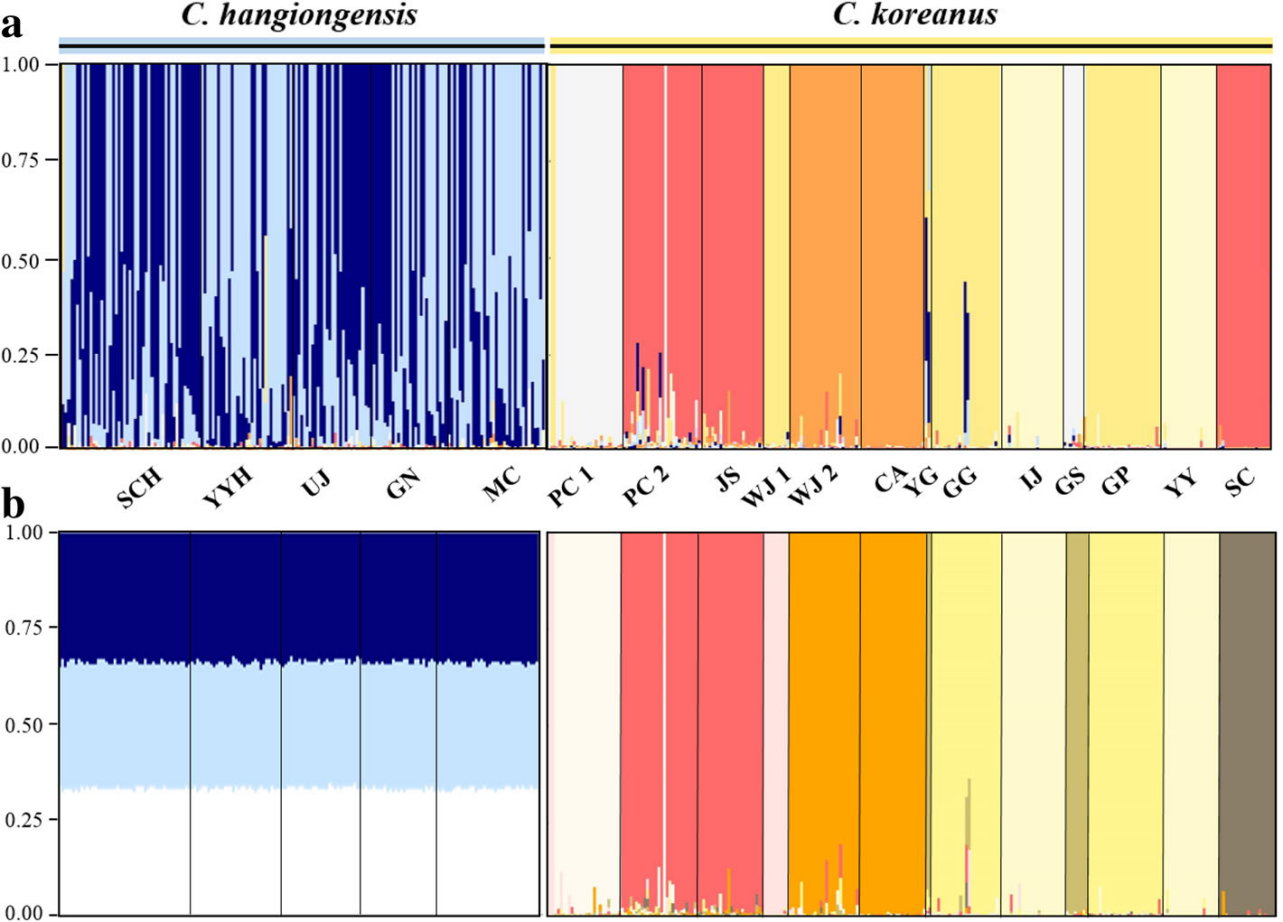
Analyses of spatial genetic structure using a Bayesian population assignment test with STRUCTURE for the five and 13 populations of *C. hangiongensis* and *C. koreanus*, respectively based on seven microsatellite loci. **a** The bar plot suggesting seven genetic clusters [*K* = 7: determined by LnP (D) = 148.671 and ΔK = 10.952] when the analysis was performed for *C. hangiongensis* and *C. koreanus* combined; **b** When the analysis was done separately for each species, all *C. hangiongensis* individuals were assigned to approximately equal proportions of the inferred three genetic clusters. However, the plot suggests eight genetic clusters [*K* = 8: LnP (D) = 220.347 and ΔK = 14.934] for *C. koreanus*. The x-axis represents each individual and the y-axis represents the probability of a given individual belonging to each of the genetic clusters. Population abbreviations as in text and Additional file 1: Table S1

STRUCTURE analysis showed that the 18 populations analysed across both species are most likely to form seven genetically distinct clusters (*K* = 7) (Fig. 4a). This number of genetic clusters was determined by: LnP (D) = 148.671 and ΔK = 10.952. Among the seven groups identified, two groups comprised populations solely of *C. hangiongensis* with a signature of high levels of genetic admixture and five clusters constituted the13 populations of *C. koreanus* (Fig. 4a). Within *C. koreanus*, only the PC 2 population showed moderate levels of genetic admixture, and the remaining populations were barely admixed (except the CA and SC populations, which consisted of a single genetic integrity). Some individuals of *C. koreanus* showed a signal of genetic introgression from *C. hangiongensis* into these individuals, although population-level admixture between the species was very low (Fig. 4a).

**Fig. 5:**
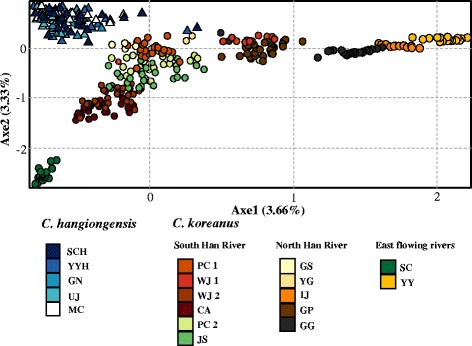
Factorial Correspondence Analysis (FCA) based on seven microsatellite genotypes for the five and 13 populations of *C. hangiongensis* and *C. koreanus*, respectively. Filled triangles represent *C. hangiongensis* individuals genotyped, and filled circles denote *C. koreanus*

When STRUCTURE analyses were performed separately for the two sculpin species, *C. hangiongensis* populations showed homogeneous distributions of individual genotypes, in which all individuals were assigned to approximately equal proportions of the inferred three genetic clusters (Fig. 4b), suggesting those populations are genetically indistinguishable. However, *C. koreanus*’ eight genetically distinct clusters (*K* = 8) best fitted the data (Fig. 4b). In this reduced analysis, *C. koreanus* populations clustered similarly to the full analysis, except that the YG, GS and SC populations, which flow into the East Sea, formed two new genetic clusters (YG and GS, and SC; Fig. 4b). The eight genetic clusters observed were determined by: LnP (D) = 220.347 and ΔK = 14.934. In addition, the second most likely *K* for *C. koreanus* was equal to 10 [LnP (D) = 941.295 and ΔK = 3.381]. Similar to the results of STRUCTURE, the FCA of seven microsatellites also revealed a single genetically indistinguishable group within *C. hangiongensis*, but every population is its own group within *C. koreanus* (Fig. 5).

## Discussion

### Differences in levels of genetic diversity between amphidromous and fluvial sculpins

We find that an amphidromous sculpin species *C. hangiongensis*, with a post-hatching larval phase has considerably higher levels of within-population genetic diversities than a fluvial landlocked species *C. koreanus* with direct development. This was true for both mtDNA control region sequences and seven microsatellite loci, although only microsatellites were statistically significant (Fig. 2). The observed higher genetic diversity in the amphidromous sculpin species, relative to the fluvial sculpin, agrees with a previous study of allozyme markers that found that populations of amphidromous *C. amblystomopsis* had greater levels of heterozygosity than those of fluvial *C. nozawae* on Hokkaido Island in Japan [3]. The elevated levels of within-population genetic diversity observed for *C. hangiongensis* might reflect their larger *N*_e_ [2, 60], which is supported by our LD-based estimates of contemporary *N*_e_ for populations of *C. hangiongensis* and *C. koreanus* (Table 2). Our *N*_e_ estimates indicate that the median *N*_e_ in *C. hangiongensis* (mean *N*_e_ = 485.23 when the SCH and MC populations [*N*_e_ = infinity] were excluded) is approximately 8.4 times greater than that in *C. koreanus* (mean *N*_e_ = 57.80 when the CA, IJ and GP populations [*N*_e_ = infinity] were excluded), providing direct evidence supporting our hypothesis.

Amphidromous *C. hangiongensis* typically produces many small eggs (752–1376 per clutch; mean = 1005) in the lower reaches of rivers [24]. Newly hatched larvae float to the surface of the river as a phototactic response and drift downstream to the river mouth [25, 33]. The planktonic larvae spend approximately one and a half months in coastal environments, in which they move with oceanic currents and return to the estuary and settle on the riverbed. This lifestyle may facilitate genetic exchange between geographically disconnected populations via larval dispersal (i.e. gene flow), at least partly contributing to the observed higher levels of within-population genetic diversity in *C. hangiongensis* [10, 61]. The home range of this amphidromous species at an adult life stage was observed to be no larger than 92 m [29]. By comparison, fluvial *C. koreanus* produces fewer but larger eggs (538–880 per clutch; mean = 744) in the upstream rivers, and hatched larvae undergo direct development and maintain a benthic life [24, 62]. In particular, the home range of this fluvial species is found to have a lifetime migration distance within 10 m, according to a transponder telemetry-based survey [28]. These findings suggest extremely low dispersal capacity for *C. koreanus* that leads to restricted gene flow among populations over geographic scales of tens to hundreds of metres. Smaller *N*_e_ along with a poorer dispersal ability makes *C. koreanus* populations more susceptible to genetic drift, resulting in the lower extent of within-population genetic diversity [63]. A previous study [5] estimated and compared the level of microsatellite diversity between amphidromous and fluvial landlocked populations within *Rhinogobius* sp. in Okinawa Island, Japan, and found much greater genetic diversity in the amphidromous populations. As such, the higher extent of genetic diversity in *C. hangiongensis* is likely attributable to high levels of gene flow and larger *N*_e_ resulting from its amphidromous life history with a pelagic larval phase. In contrast, the low genetic diversity in *C. koreanus* is presumed to be due to genetic isolation among individual populations resulting from its fluvial landlocked life history with direct development. The greater fecundity (approximately 1.4 times higher fecundity) of *C. hangiongensis* may also contribute to its higher intraspecific genetic variation [2]. Our results also imply that while genetic variation from samples at a locality in *C. hangiongensis* may represent a complete picture of the genetic diversity due to high gene flow from surrounding sites, genetic variation in *C. koreanus* may be partitioned among different localities due to very low gene flow [64].

Alternatively, amphidromous and fluvial species have undergone very different demographic population histories during the last glacial maximum, as sea level fluctuations could have affected these species differently [65]. If glaciations had an effect on genetic diversity, we would expect to see large population bottlenecks for both species. However, this is not true for either species, given that only one (CA) of ten populations in *C. koreanus* and none of the populations in *C. hangiongensis* showed evidence for population bottleneck (Table S2). Therefore, the observed differences in within-population genetic diversity between these species can be most likely attributed to their differing early life histories. Most populations of both species appear to be in HWE, except for the SCH population of *C. hangiongensis* and the PC 1, JS, IJ, GP and GG populations of *C. koreanus*, which suggests non-random mating at the loci tested. Somewhat ironically, the SCH population of amphidromous *C. hangiongensis* shows a genetic signal of inbreeding, albeit weak, whereas the IJ and GP populations of fluvial *C. koreanus* show a detectable signal of outbreeding (Table 1). In theory, small *N*_e_ is likely to result in high rates of inbreeding, causing an increase in homozygosity [66]. Nevertheless, the limited dispersal and reported polygynous mating system of the fluvial sculpin may account for outbreeding within the IJ and GP populations. After spawning, the male chases the female partner away from its nest, while guarding the eggs and embryos [27]. Our finding that a critically endangered species, *C. hangiongensis*, has high genetic diversity is fairly good news for conservation and its future persistence. None of the five populations of this species analysed shows a genetic signal of a population bottleneck. On the other hand, *C. koreanus* might be more threatened than previously thought, as it shows lower levels of genetic diversity and limited connectivity when compared to *C. hangiongensis*.

### Differences in patterns of population connectivity and spatial population structure between amphidromous and fluvial sculpins

Our results from both mtDNA and microsatellite markers reveal noticeable differences in the magnitude and patterns of population connectivity between the two sculpin species that vary in the presence of a pelagic larval phase. Amphidromous *C. hangiongensis*, with a planktonic larval period, shows apparently less spatial population structure relative to the fluvial *C. koreanus*, with a benthic lifestyle. The lower spatial genetic variation of the amphidromous species is most likely due to the greater larval dispersal than the fluvial species, given their restricted ranges as adults (tens of metres). The magnitude of spatial genetic differentiation, as suggested by pairwise *F*-statistics, was markedly lower within *C. hangiongensis* than within *C. koreanus* (Table 3). The *F*_ST_ values for *C. koreanus* were all highly significant, which is consistent with a previous population genetics study of this species with AFLP (Amplified Fragment Length Polymorphism) marker [39]. These results suggest that gene flow is restricted among the 10 populations of *C. koreanus* examined, and that this is most likely caused by limited dispersal ability. A recent study of the Manchurian trout *Brachymystax lenok tsinlingensis* from South Korea inhabiting watersheds that overlap like those of *C. koreanus* also showed significant genetic divergence among populations [67]. Examining another species pair in the genus *Cottus* that differs in the presence/absence of the post-hatching larval stage on Hokkaido Island in Japan, a previous study [3] also demonstrated that the amphidromous *C. amblystomopsis* populations are less structured than the fluvial *C. nozawae*. These previous findings also support the hypothesis that the divergent patterns of genetic structure between *C. hangiongensis* and *C. koreanus* may have been caused by differences in life history traits.

IBD analysis reveals no correlation between geographic and genetic distances among the populations in both species, indicating that the geographic proximity of populations is not responsible for the observed spatial population structure of either species. For amphidromous *C. hangiongensis*, geographic distance among localities does not seem to play a role in shaping the population structure over the geographical scales analysed (9–140 km), perhaps due to the effects of connectivity during larval phases promoted by the oceanic currents. However, a study of short ninespine stickleback *Pungitius kaibarae* without a larval phase at the east-flowing river basins in South Korea revealed strong inter-population genetic differentiation [68], which supports the notion that the absence of genetic structuring in *C. hangiongensis* is highly likely due to the presence of the larval phase. The lack of population structure (i.e. high population connectivity) observed for *C. hangiongensis* suggests that the juveniles of this species do not necessarily return to their home rivers where they hatched. For fluvial *C. koreanus*, its very low dispersal ability may mask the role of geographic distance that contributes to the population structure over the geographic scales we tested (1–177 km). Further studies on smaller geographic scales will be required to determine the spatial scales at which *C. koreanus* populations are structured.

The evidence that amphidromous *C. hangiongensis* comprises a single random mating population, but fluvial *C. koreanus* consists of genetically distinct individual populations is further supported by the results of our STRUCTURE and FCA analyses. While genotyped individuals of *C. hangiongensis* from five localities represent a single population with a high degree of admixture, *C. koreanus* individuals from thirteen sites were divided into five (or eight) genetically unique clusters (Fig. 4). However, the genetic groups within *C. koreanus* do not correspond well to river basins, such as the South Han River, the North Han River and east-flowing river basins, as suggested in a previous study of the Korean lenok [67].

Given the similarity of traits such as diet composition [37, 38], mating system [27, 69], spawning season [27, 69] and adult’s home range [28, 29] between the two species, it is highly likely that the observed differences in the magnitude and pattern of genetic diversity and geographic population structure between *C. hangiongensis* and *C. koreanus* is driven by their divergent life histories [3, 5]. These closely related species are very similar to each other, to the point where they are not fully ecologically or genetically isolated. We found evidence for hybridization on our microsatellite dataset, and we will evaluate the extent and outcomes of these events in future research projects by applying ecological and genomic approaches.

## Conclusions

This study suggests that an amphidromous species with a planktonic larval phase, *C. hangiongensis*, shows higher levels of within-population genetic diversity than a fluvial species with direct development, *C. koreanus*. On the contrary, the fluvial sculpin exhibits much greater levels of geographic population structure than the amphidromous sculpin. While *C. hangiongensis* represents a single genetically homogeneous population, *C. koreanus* reflects genetically distinct local populations. We conclude that contrasting early life histories – including the presence or absence of the larval phase – may have led to divergent patterns of genetic diversity, population connectivity and spatial population structure between these two sister-species of sculpin. This study highlights the potentially important role of life history traits in the evolution of population genetic structure and suggests the need for further investigations into how life history features contribute to speciation more generally.

## Additional file

Additional file 1: **Table S1.** Information of sampling localities, population codes, coordinate (latitude/longitude), and river basins. **Table S2.** Statistical tests for a recent bottleneck in each of the five and 10 populations of *C. hangiongensis* and *C. koreanus*, respectively from South Korea. *P*-values are based on the Wilcoxon test. Allelic frequency distribution shape was normal or shifted for mode-shift distortion. Population abbreviations as in text and Additional file 1: **Table S1**. (ZIP 26 kb)

## Abbreviations

AFLP: Amplified fragment length polymorphism
AR: Allelic richness
CA: Chiaksan National Park
CI: Confidence interval
CR: Control regions
FCA: Factorial correspondence analysis
*F*_IS_: Inbreeding coefficient
GG: Pocheon
GN: Gangneung
GP: Gapyeong
GS: Goseong
*h*: Haplotype diversity
*H*_E_: Expected heterozygosity
*H*_O_: Observed heterozygosity
HR: Haplotype richness
HWE: Hardy-Weinberg equilibrium
IBD: Isolation by distance
IJ: Inje
JS: Jeongseon
LD: Linkage disequilibrium
MC: Mulchi
MtDNA: Mitochondrial DNA
*N*_A_: Mean number of alleles per locus
*N*_e_: Effective population sizes
OK: Okgye
PA: Private alleles
PC 1: Pyeongchang from the Heungjeong Valley
PC 2: Pyeongchang from the Gihwa Stream
PCR: Polymerase chain reaction
SC: Samcheok (for *C. koreanus*)
SCH: Samcheok (for *C. hangiongensis*)
TPM: Two-phase mutation
UJ: Uljin
WJ 1: Wonju Oakvalley
WJ 2: Wonju from the Gangrim Stream
YG: Yanggu
YY: Yangyang (for *C. koreanus*)
YYH: Yangyang (for *C. hangiongensis*)
π: Nucleotide diversity
