# Supplementary material for: Contrasting life histories contribute to divergent patterns of genetic diversity and population connectivity in freshwater sculpin fishes
Source: BMC Evol Biol. 2018 Apr 11;18:52. doi: 10.1186/s12862-018-1171-8 (PMC5896141; doi:10.1186/s12862-018-1171-8)
Supplement: Supplementary file 1 — Table S1. Information of sampling localities, population codes, coordinate (latitude/longitude), and river basins. Table S2. Statistical tests for a recent bottleneck in each of the five and 10 populations of C. hangiongensis and C. koreanus, respectively from South Korea. P-values are based on the Wilcoxon test. Allelic frequency distribution shape was normal or shifted for mode-shift distortion. Population abbreviations as in text and Additional file 1: Table S1. (ZIP 26 kb) [file 12862_2018_1171_MOESM1_ESM.zip › Table S1. Information of sampling localities .docx]

**Table S1** Information of sampling localities, population codes, coordinate (latitude/longitude), and river basins.

|  | Location | Population code | Latitude | Longitude | River basin |
| --- | --- | --- | --- | --- | --- |
| *C. hangiongensis* | Yangyang | YYH | 38°4'15"N | 128°37'25"E | East flowing rivers |
|  | Mulchi | MC | 38°09'05"N | 128°36'10"E | East flowing rivers |
|  | Gangneung | GN | 37°51'7"N | 128°49'52"E | East flowing rivers |
|  | Okgye | OK | 37°62'48"N | 129°03'44"E | East flowing rivers |
|  | Samcheok | SCH | 37°25'59"N | 129°07'35"E | East flowing rivers |
|  | Uljin | UJ | 36° 57'58"N | 129°23'45"E | East flowing rivers |
| *C. koreanus* | Goseong | GS | 38°29'53"N | 128°23'48"E | North Han River |
|  | Yanggu | YG | 38˚15'28"N | 128˚02'47"E | North Han River |
|  | Inje | IJ | 38°01'44"N | 128°28'20"E | North Han River |
|  | Gapyeong | GP | 37°51'1"N | 127°20'19"E | North Han River |
|  | Pocheon | GG | 37°44'52"N | 129°09'46"E | North Han River |
|  | Pyeongchang from the Heungjeong Valley | PC 1 | 37˚38.98'N | 128˚19.67'E | South Han River |
|  | Pyeongchang from the Gihwa Stream | PC 2 | 37°18'31"N | 128°31'48"E | South Han River |
|  | Jeongseon | JS | 37°22'24"N | 128°42'42"E | South Han River |
|  | Wonju Oakvalley | WJ 1 | 37°23'13"N | 127°50'33"E | South Han River |
|  | Wonju from the Gangrim Stream | WJ 2 | 37°19.52"N | 128°04.41"E | South Han River |
|  | Chiaksan National Park | CA | 37°20'01"N | 128°04'18"E | South Han River |
|  | Samcheok | SC | 37°16'31"N | 129°04'21"E | East flowing rivers |
|  | Yangyang | YY | 37˚54'44"N | 128˚29'18"E | East flowing rivers |
