# Supplementary material for: Contrasting life histories contribute to divergent patterns of genetic diversity and population connectivity in freshwater sculpin fishes
Source: BMC Evol Biol. 2018 Apr 11;18:52. doi: 10.1186/s12862-018-1171-8 (PMC5896141; doi:10.1186/s12862-018-1171-8)
Supplement: Supplementary file 1 — Table S1. Information of sampling localities, population codes, coordinate (latitude/longitude), and river basins. Table S2. Statistical tests for a recent bottleneck in each of the five and 10 populations of C. hangiongensis and C. koreanus, respectively from South Korea. P-values are based on the Wilcoxon test. Allelic frequency distribution shape was normal or shifted for mode-shift distortion. Population abbreviations as in text and Additional file 1: Table S1. (ZIP 26 kb) [file 12862_2018_1171_MOESM1_ESM.zip › Table S2. Bottleneck analysis.docx]

**Table S2** Statistical tests for a recent bottleneck in each of the five and 10 populations of *C. hangiongensis* and *C. koreanus*, respectively from South Korea. *P*-values are based on the Wilcoxon test. Allelic frequency distribution shape was normal or shifted for mode-shift distortion. Population abbreviations as in text and Table S1.

| Species | Population | Deficient/excess | *P*-value | Distribution shape |
| --- | --- | --- | --- | --- |
| *C*. *hangiongensis* | SCH | 5/2 | 0.078 | Normal |
|  | YYH | 5/2 | 0.078 | Normal |
|  | UJ | 4/3 | 0.109 | Normal |
|  | GN | 4/3 | 0.468 | Normal |
|  | MC | 4/3 | 0.109 | Normal |
| *C*. *koreanus* | PC 1 | 3/4 | 1.000 | Normal |
|  | PC 2 | 2/5 | 0.938 | Normal |
|  | WJ 2 | 5/2 | 0.078 | Normal |
|  | CA | 3/4 | 0.469 | Shifted |
|  | JS | 3/4 | 0.938 | Normal |
|  | SC | 3/2 | 0.156 | Normal |
|  | YY | 2/1 | 1.000 | Normal |
|  | IJ | 2/2 | 0.313 | Normal |
|  | GP | 2/5 | 0.078 | Normal |
|  | GG | 4/3 | 0.375 | Normal |
